# Induction of diabetes by Tacrolimus in a phenotypic model of obesity and metabolic syndrome

**DOI:** 10.3389/fendo.2024.1388361

**Published:** 2024-04-29

**Authors:** Silvia Teixidó-Trujillo, Esteban Porrini, Luis Manuel Menéndez-Quintanal, Armando Torres-Ramírez, Cecilia Fumero, Ana Elena Rodríguez-Rodríguez

**Affiliations:** ^1^Facultad de Medicina, Universidad de La Laguna, San Cristóbal de La Laguna, Santa Cruz de Tenerife, Spain; ^2^Research Unit, Hospital Universitario de Canarias, San Cristóbal de La Laguna, Santa Cruz de Tenerife, Spain; ^3^Instituto de Tecnologías Biomédicas (ITB), Universidad de la Laguna, San Cristóbal de La Laguna, Santa Cruz de Tenerife, Spain; ^4^Department of Chemistry and Drugs, National Institute of Toxicology and Forensic Sciences, San Cristóbal de La Laguna, Santa Cruz de Tenerife, Spain; ^5^Nephrology Department, Hospital Universitario de Canarias, San Cristóbal de La Laguna, Santa Cruz de Tenerife, Spain

**Keywords:** type 2 diabetes, post-transplant diabetes, Tacrolimus, obesity, metabolic syndrome, animal model

## Abstract

**Introduction:**

The pathogenesis of Post-Transplant Diabetes Mellitus (PTDM) is complex and multifactorial and it resembles that of Type-2 Diabetes Mellitus (T2DM). One risk factor specific to PTDM differentiates both entities: the use of immunosuppressive therapy. Specifically, Tacrolimus interacts with obesity and insulin resistance (IR) in accelerating the onset of PTDM. In a genotypic model of IR, the obese Zucker rats, Tacrolimus is highly diabetogenic by promoting the same changes in beta-cell already modified by IR. Nevertheless, genotypic animal models have their limitations and may not resemble the real pathophysiology of diabetes. In this study, we have evaluated the interaction between beta-cell damage and Tacrolimus in a non-genotypic animal model of obesity and metabolic syndrome.

**Methods:**

Sprague Dawley rats were fed a high-fat enriched diet during 45 days to induce obesity and metabolic dysregulation. On top of this established obesity, the administration of Tacrolimus (1mg/kg/day) during 15 days induced severe hyperglycaemia and changes in morphological and structural characteristics of the pancreas.

**Results:**

Obese animals administered with Tacrolimus showed increased size of islets of Langerhans and reduced beta-cell proliferation without changes in apoptosis. There were also changes in beta-cell nuclear factors such as a decrease in nuclear expression of MafA and a nuclear overexpression of FoxO1A, PDX-1 and NeuroD1. These animals also showed increased levels of pancreatic insulin and glucagon.

**Discussion:**

This model could be evidence of the relationship between the T2DM and PTDM physiopathology and, eventually, the model may be instrumental to study the pathogenesis of T2DM.

## 1 Introduction

Post-transplant diabetes mellitus (PTDM) is frequent following solid organ transplantation and affects about 30% of the renal transplant population. This condition is associated with a higher rate of patient mortality, cardiovascular disease, cancer and infection (1–3).

The pathogenesis of PTDM is complex and multifactorial. Risk factors of PTDM are similar to those for type-2 diabetes mellitus such as obesity, metabolic syndrome (MS) and insulin resistance (IR) (2, 4–6). However, there is one factor specific of PTDM, which is the use of immunosuppressant medications like steroids, Cyclosporin, Tacrolimus, Sirolimus and Everolimus. Of them, Tacrolimus (TAC) is the most commonly used immunosuppressant (5). In obese patients with metabolic syndrome and insulin resistance TAC presents a particularly high diabetogenic effect (5, 6). Nowadays, TAC can be considered one of the main causes of PTDM. Thus, understanding beta-cell toxicity by TAC is necessary to understand PTDM. In previous studies our group has reported that TAC accelerates the transition from pre-diabetes to diabetes in a genotypic model of leptin receptor-deficient obese Zucker rats. This effect is caused by the TAC action on the same beta-cell damage pathways already modified by IR. Some of these affected pathways are transcription factors crucial for the correct behaviour of beta cells like MafA, FoxO1A, PDX-1 and NeuroD1 (1, 7). This action of TAC on top of cells affected by IR may indicate common links between the pathogenesis of diabetes and PTDM.

Genotypic and spontaneous animal models like Zucker rats have been extensively used in diabetes research, and they have both advantages and disadvantages. Genotypic models have a known homogeneous genetic background (8, 9) where diabetes develops spontaneously with standard diet ad libitum. This avoids time-consuming feeding schemes and invasive procedures, like partial pancreatectomy, a risk for the integrity of the animal (10–12). Altogether these characteristics make the results more reproducible (8). However, most of these animal models are caused by monogenic mutations which represent 2-5% of diabetes in humans (8, 13). Clearly, the clinical heterogeneity of diabetes may not be explained by a single mutation (13). For example, in diabetic patients, leptin receptor mutations are quite infrequent (11), and therefore genotypic animal models may not resemble the real pathophysiology of diabetes (11, 14, 15).

Although genotypic animal models may have their utility as a proof of concept in basic diabetic research, a shift to phenotypic-based diabetes models that resemble more clearly the pathophysiology of the disease has been recently recommended (11).

In this study we have evaluated the interaction between beta-cell damage and TAC toxicity in a non-genotypic animal model of Sprague Dawley rats. For this purpose, metabolic syndrome was induced by feeding the animals a high fat diet, and PTDM was accelerated by the administration of Tacrolimus during 15 days. We evaluated changes in glucose metabolism, insulin resistance, modifications in transcription factors essentials for beta cell functionality and identity (MafA, FoxO1A, PDX-1 and NeuroD1) as well as changes in the content of insulin and glucagon.

## 2 Material and methods

### 2.1 Animal model

A total of 25 male Sprague Dawley rats of approximately 8 weeks old were used for this study. Animals were housed in cages at constant temperature (22°C) with a 12:12h light-dark cycle and relative humidity of 50% in the animal house of the University of La Laguna (ULL). Animal care was performed in accordance with institutional guidelines in compliance with Spanish (Real Decreto 53/2013, February 1. BOE, 8 February 8, 2013, n: 34, p. 11370-11421) and international laws and policies (Directive 9 2010/63/EU of the European Parliament and of the Council of 22 September 2010 on the protection of animals used for scientific purposes) and the ARRIVE guidelines on the care and use of animals for scientific purposes, and were approved by the Institutional Animal Care and Use Committee (Comité de Ética de la Investigación y de Bienestar Animal (CEIBA) of University of La Laguna, Spain).

### 2.2 Experimental design

#### 2.2.1 Induction of metabolic syndrome

The 25 Sprague Dawley rats were randomized in two groups: High Fat Diet (HFD) to induce obesity and MS (n=14) and Standard Diet (SD) as a control (n=11). Animals were fed ad libitum water and each dietary regimen during 45 days [dietary duration adapted from Zhang Y et al. (16)] (**Supplementary Figure 1**). Animals on HFD received a purified diet (Research Diets, D12492) in which calories are derived from proteins (20%), carbohydrates (20%) and fat (60%), including 232 mg cholesterol (lard and blue dye); for a total of 5.24 kcal per gram. Animals on SD received a non-purified commercial diet (Teklad global diet, Envigo) in which calories are from proteins (20%), carbohydrates (67%) and fat (13%).

#### 2.2.2 Induction of PTDM

After 45 days of diet, animals in each group were randomized to receive a daily dose of Tacrolimus (Prograf 5mg/ml) 1 mg/kg (7 on HFD and 6 on SD) or Vehicle (7 on HFD and 5 on SD) injected intraperitoneally during 15 days (**Supplementary Figure 1**). Animals were weighted 5 times a week. At the end of the experiment animals were sacrificed by intraperitoneal injection of pentobarbital (75 mg/kg). Blood was collected and serum separated. Pancreases were collected, fixated in 4% formaldehyde for 24h and paraffin- embedded.

### 2.3 Procedures

At baseline, after 45 days of each dietary regimen and at the end of the experiment, animals underwent the following procedures to determine their biochemical values.

#### 2.3.1 Intra-peritoneal glucose tolerance test

After 12h of fasting, glucose (2g/kg) was injected and blood glucose was measured at 0, 5, 15, 30, 60 and 120 min with an Accu-Check glucometer. Hyperglycaemia was defined as 120-min glycaemia in the IPGTT>200 mg/dL.

#### 2.3.2 Insulin tolerance test

Non-fasting animals were injected with 0,75 U/kg of insulin and blood glucose was measured at 0, 5, 15, 30, 60 and 120 min with an Accu-Check glucometer. The area under the curve (AUC) of each animal was calculated to evaluate insulin resistance.

### 2.4 Biochemical parameters

Serum insulin levels were measured at the endpoint using an ELISA kit (enzyme-linked immunosorbent assay) (Mercodia, Sweden).

### 2.5 Tacrolimus pharmacokinetic

After 7 days of TAC administration, the compound pharmacokinetic was determined to corroborate animals receiving appropriate doses of CNI similar to those used in clinical practice (8-10 ng/mL). Briefly, once the Tacrolimus was administered, blood samples (20 μl) were collected from the tip of the tail with heparinized capillary tubes (Hirschmann) before and 5, 15, 60 min, 6h and 24h after injection. Blood samples were deposited in Eppendorf tubes and refrigerated until analysis. Then, TAC whole blood concentration was measured by liquid chromatography-high resolution mass spectrometry (LC-HRMS Orbitrap) according to the previously published methods (17, 18).

### 2.6 Determination of nuclear factors expression, islet morphometry, proliferation and apoptosis

The expression of MafA, FoxO1A, PDX-1, NeuroD1, Insulin, Glucagon and Ki67 in pancreas tissue was evaluated by immunofluorescence. Beta cells were identified using Nkx6.1 (19). The area of the islets of Langerhans and the proportion of alpha and beta cells were quantified. Pancreas cross-sections of three-micron thick were deparaffinized and rehydrated. Tissue sections were immersed into Target Retrieval Solution for 15 minutes at 95°C for antigen retrieval. Slides were incubated with PBS 1X + 0.1% Triton X-100 for the membranes permeabilization and blocked with PBS 1X + BSA 3% + 0.1% azida. Primary antibody was added to the tissues as follows: MafA (Abcam, ab26405, 1:500), FoxO1A (Abcam, ab52857, 1:250), PDX-1 (Abcam, ab47267, 1:250), NeuroD1 (Abcam, ab60704, 1:250), Nkx6.1 (BD Pharmingen, 563022, 1:500) NKx6.1 (Abcam, ab221549, 1:250), Insulin (Invitrogen, 18-0067, 1:500), Glucagon (Abcam, ab92517, 1:500), ki67 (Abcam, ab15580, 1:500). The slides were incubated with the primary antibody overnight in a humidified chamber at 4°C. Then, sections were incubated with secondary antibodies for 1h at room temperature (AlexaFluor-488 Goat Anti-Rabbit IgG (1:1000), AlexaFluor-555 Goat Anti-Mouse IgG (1:1000), and AlexaFluor-594 Goat Anti-Guinea pig IgG (1:1000)). Section were incubated 5 minutes with 4,6-Diamidino-2-phenylindole (DAPI) (Invitrogen) and mounted with PBS1X:Glicerol 1:1. Slides were observed using a Zeiss fluorescent microscope and photographed with ZEN imaging software. For quantitative analysis, ten to twelve images per animal were acquired under the 20-fold microscope. MafA, FoxO1A, PDX-1 and NeuroD1 fluorescence was quantified as nuclear Integrated Density in double positive nuclei cells (MafA+/FoxO1A+/PDX-1+/NeuroD1+, Nkx6.1+). Insulin and Glucagon fluorescence was quantified as cytoplasmic Integrated Density in the Langerhans’ Islet. The positive signal of the different markers was quantified using Image J software (National Institute of Health). An average of the positive signal of all images for each group was determined for analysis.

### 2.7 Statistical analysis

Statistical analysis was performed with GraphPad Prism 8 (GraphPad Software Inc, San Diego, CA) and IBM SPSS Statistics 20 (Chicago, IL). Data are expressed as mean ± standard deviation. To compare groups, one-way ANOVA or two-way ANOVA were used and statistical significance was considered at p<0.05. Mann-Whitney test was used in non-parametric comparison and statistical significance was considered at p<0.05.

## 3 Results

### 3.1 Induction of metabolic syndrome

After 45 days of SD or HFD, only those animals on HFD developed metabolic syndrome.

#### 3.1.1 Body weight

**Figure 1:**
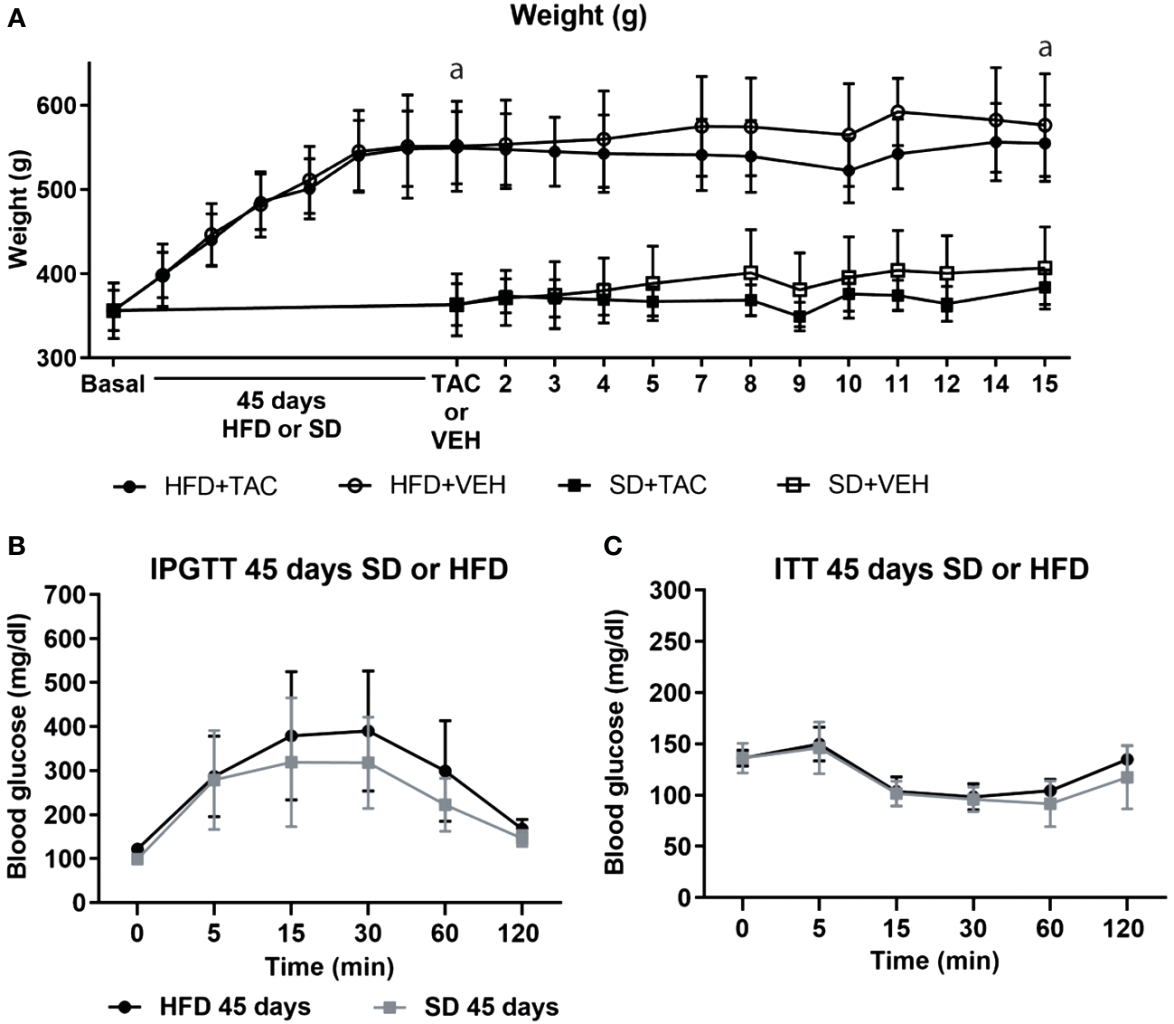
Metabolic characteristic of Sprague Dawley rats after 45 days of HFD or SD feeding. **(A)** Animal weight increased during all of the experiment (a – HFD animal vs. SD animal p ≤ 0.0081). **(B)** Intra-peritoneal glucose tolerance test (IPGTT) after 45 days on HFD or SD **(C)** Insulin tolerance test (ITT) after 45 days on HFD or SD.

Animals on HFD developed obesity after 45 days, reaching a body weight of 550.29 ± 46.7 g while animals in SD had a body weight of 374 ± 28.2 g (p ≤ 0.0081) (**Figure 1A**). Animals on HFD increase 77.2% their body weight and animals on SD 51.6%. HFD groups presented a body weight gain of 25,6% more than animals in SD.

#### 3.1.2 Glucose tolerance

In the IPGTT, animals on HFD showed higher levels of fasting blood glucose (121.6 ± 10.4 mg/dL) than those in SD (98 ± 10.8 mg/dL), although the difference was no significant (**Figure 1B**). The AUC was comparable between groups (**Supplementary Figure 2A**).

#### 3.1.3 ITT

Insulin resistance was comparable between groups in animals on HFD or SD (**Figure 1C**). The AUC was comparable between groups (**Supplementary Figure 2A**).

### 3.2 Induction of PTDM by the administration of TAC

After the induction of metabolic syndrome, 1 mg/kg of TAC or VEH was administered during consecutives 15 days. The following changes were observed:

#### 3.2.1 Body weight

No major changes in body weight were observed in all groups. Animals in HFD with or without TAC remained more obese compared to those in SD (p ≤ 0.0081) (HFD+TAC: 556.3 ± 46.0 g; HFD+VEH: 582.6 ± 62.3 g; SD+TAC: 384.0 ± 22.4 g; SD+VEH: 402.0 ± 43.7 g) (**Figure 1A**). Animals on HFD increase 79.4% their body weight and animals on SD 55.7%. HFD groups presented a body weight gain of 23,6% more than animals in SD.

#### 3.2.2 Glucose tolerance

**Figure 2:**
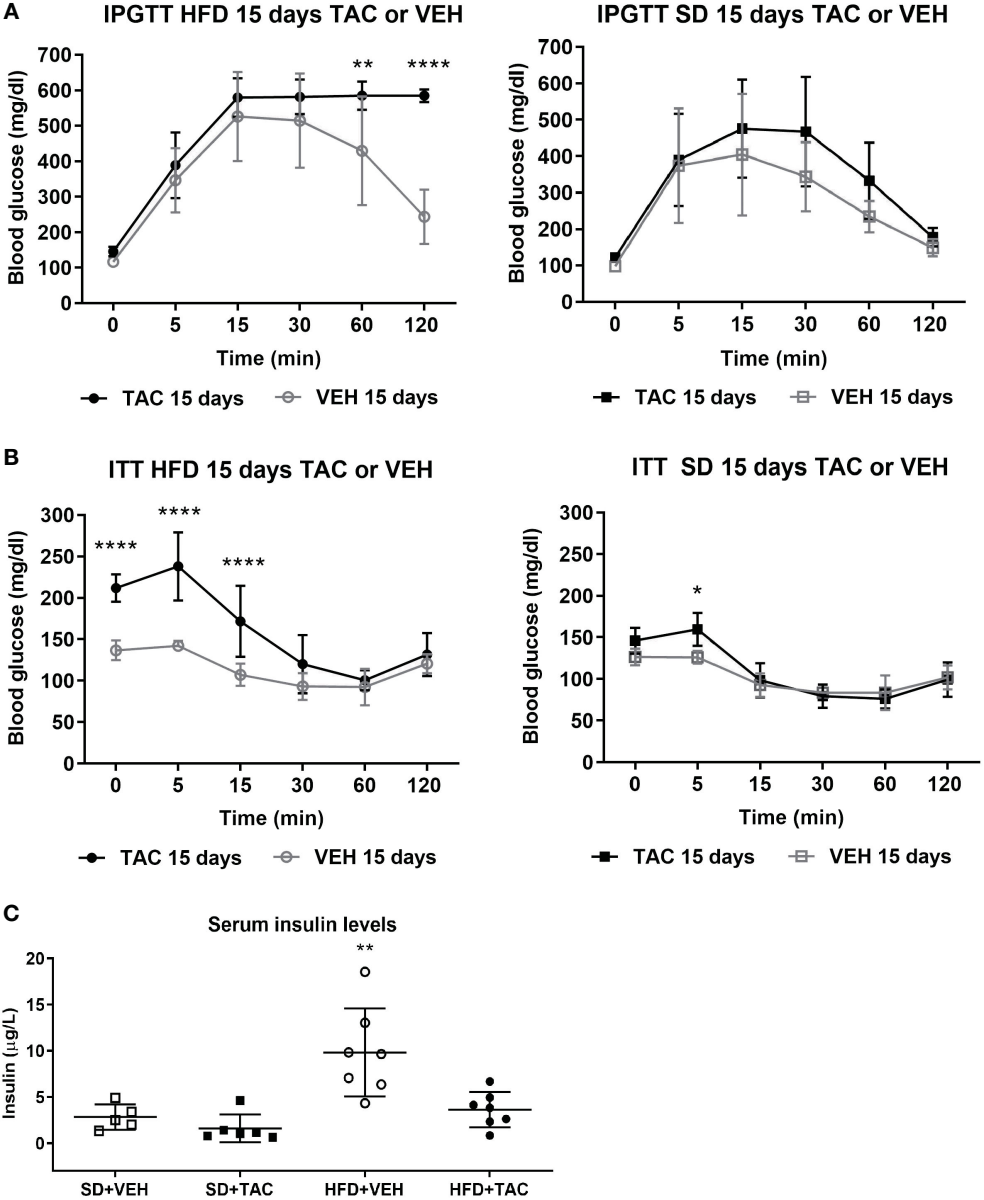
Metabolic characteristic of Sprague Dawley rats after 45 days on HFD or SD feeding and 15 days on TAC or VEH administration. **(A)** Intra-peritoneal glucose tolerance test of HFD and SD group at 15 days of TAC or VEH treatment (120 min glycaemia HFD+TAC vs. HFD+VEH p ≤ 0.0001). **(B)** Insulin tolerance test of HFD and SD group at 15 days of TAC or VEH treatment (0 min glycaemia HFD+TAC vs. HFD+VEH p ≤ 0.0001). **(C)** Serum insulin levels at endpoint (HFD+VEH vs. HFD+TAC, SD+TAC, SD+VEH p ≤ 0.0026).* - HFD+TAC vs. HFD+VEH (p≤ 0.005), ** - HFD+TAC vs. HFD+VEH (p≤ 0.0026), **** - HFD+TAC vs. HFD+VEH (p≤ 0.0001).

In obese animals, TAC induced severe glucose intolerance from 15 to 120 min in IPGTT reaching blood glucose levels of 600 mg/dL in IPGTT at 120 min (p ≤ 0.0001 vs. HFD+VEH) (**Figure 2A**). Animals in SD (with TAC or VEH) did not have hyperglycaemia in IPGTT (≤200 mg/dL) (**Figure 2A**). The AUC was higher in animals on HFD+TAC (2500 ± 212,5) compared to those on HFD+VEH (1996 ± 516,7) (p ≤ 0.011) (**Supplementary Figure 2B**).

#### 3.2.3 ITT

Animals on HFD+TAC had higher glucose levels at baseline than those on HFD+VEH group (211.9 ± 16.6 mg/dL vs. 136.7 ± 9 mg/dL p ≤ 0.0001) (**Figure 2B**). The AUC was higher in animals on HFD+TAC compared to those on HFD+VEH (802 ± 135.09 vs 563.36 ± 24.63 p ≤ 0.0006) (**Supplementary Figure 2C**). No major differences were observed in the ITT in animals on SD+TAC or SD+VEH (**Figure 2B**).

#### 3.2.4 Serum insulin levels

Animals on HFD+VEH showed higher levels of insulin compared to those on HFD+TAC (9.84 ± 4.76 µg/L vs. 3.63 ± 1.91 µg/L p ≤ 0.0026), animals on SD+VEH (2.83 ± 1.37 µg/L p ≤ 0.0026) and those on SD+TAC (1.61 ± 1.49 µg/L p ≤ 0.0026) (**Figure 2C**).

### 3.3 Pharmacokinetic and blood levels of Tacrolimus

After 24 h TAC administration (1 mg/Kg), animals on HFD showed higher levels of the drug (8.33 ± 3.1 ng/mL) than those in SD (4.16 ± 1.1 ng/mL), although the differences were not significant (**Supplementary Figure 3A**). The analysis of the pharmacokinetic curves are shown in Supplementary material (**Supplementary Figure 3B**).

### 3.4 Islet morphometry, nuclear factors expression, insulin, glucagon, proliferation and apoptosis

#### 3.4.1 Islet morphology of pancreatic tissue

Animals on HFD+TAC had higher relative islet area (41319.1 ± 24238.8 pixel/µm) than the other groups (HFD+VEH: 32210.2 ± 22007.6 pixel/µm; SD+TAC: 32636.7 ± 19325.7 pixel/µm; SD+VEH: 30376.54 ± 19600.77 pixel/µm; p ≤ 0.0005 (**Supplementary Figure 4A**).

#### 3.4.2 Nuclear factors expression

**Figure 3:**
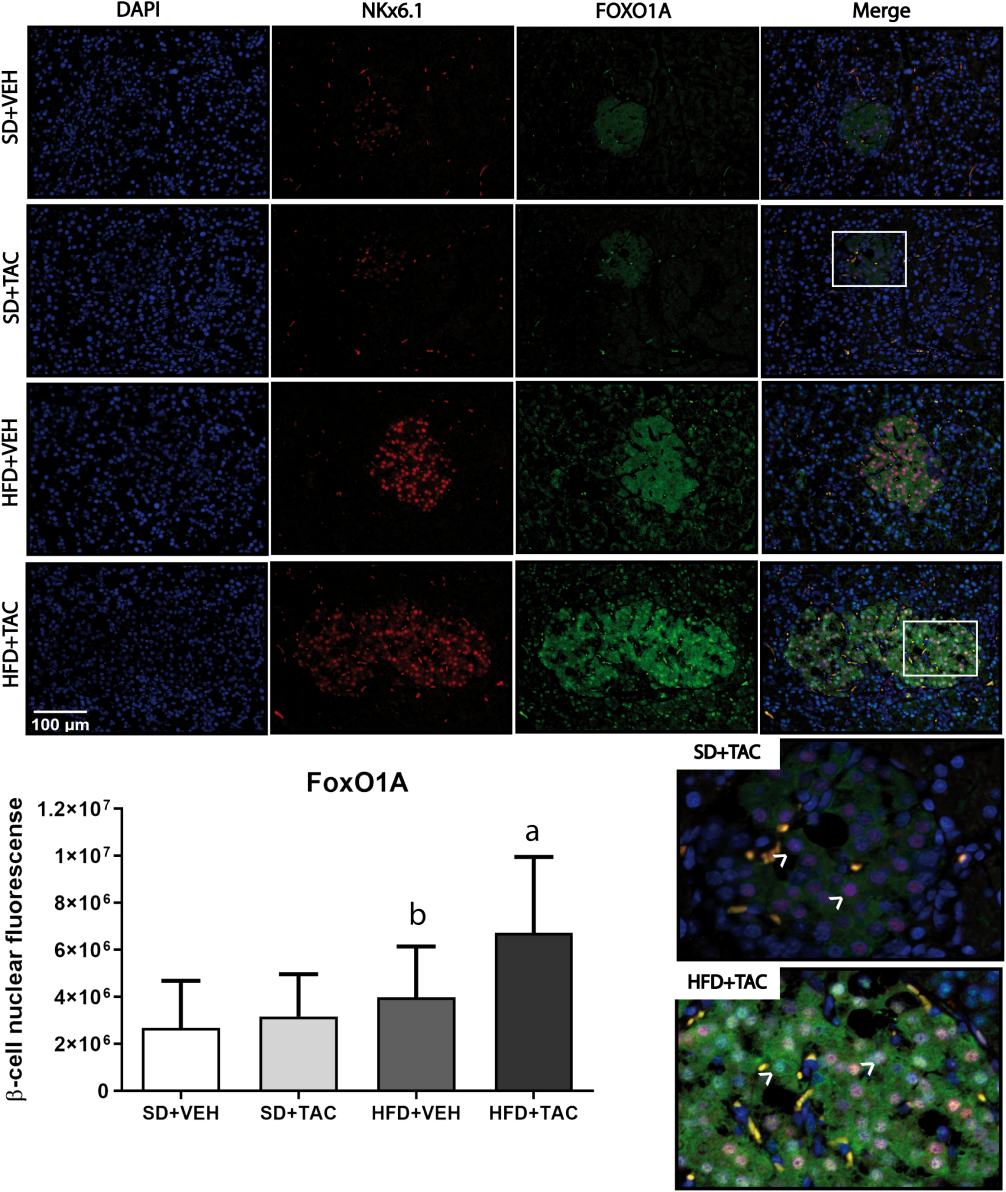
Forkhead box protein O1 (FoxO1) expression by immunofluorescence staining of Langerhans islets of pancreatic tissue and quantitative analysis. There was a marked increase in the expression of nuclear forkhead box protein O1 (FoxO1) of beta cells in pancreas of both HFD+TAC and HFD+VEH group. Data are expressed as mean plus or minus standard deviation. White arrows point the lack of expression of nuclear FoxO1 in SD+TAC and the overexpression in HFD+TAC (a - HFD+TAC vs. HFD+VEH p ≤ 0.0001; HFD+TAC vs. SD+TAC p ≤ 0.0001; HFD+TAC vs. SD+VEH p ≤ 0.0001; b - HFD+VEH vs. SD+VEH p ≤ 0.0045).

**Figure 4:**
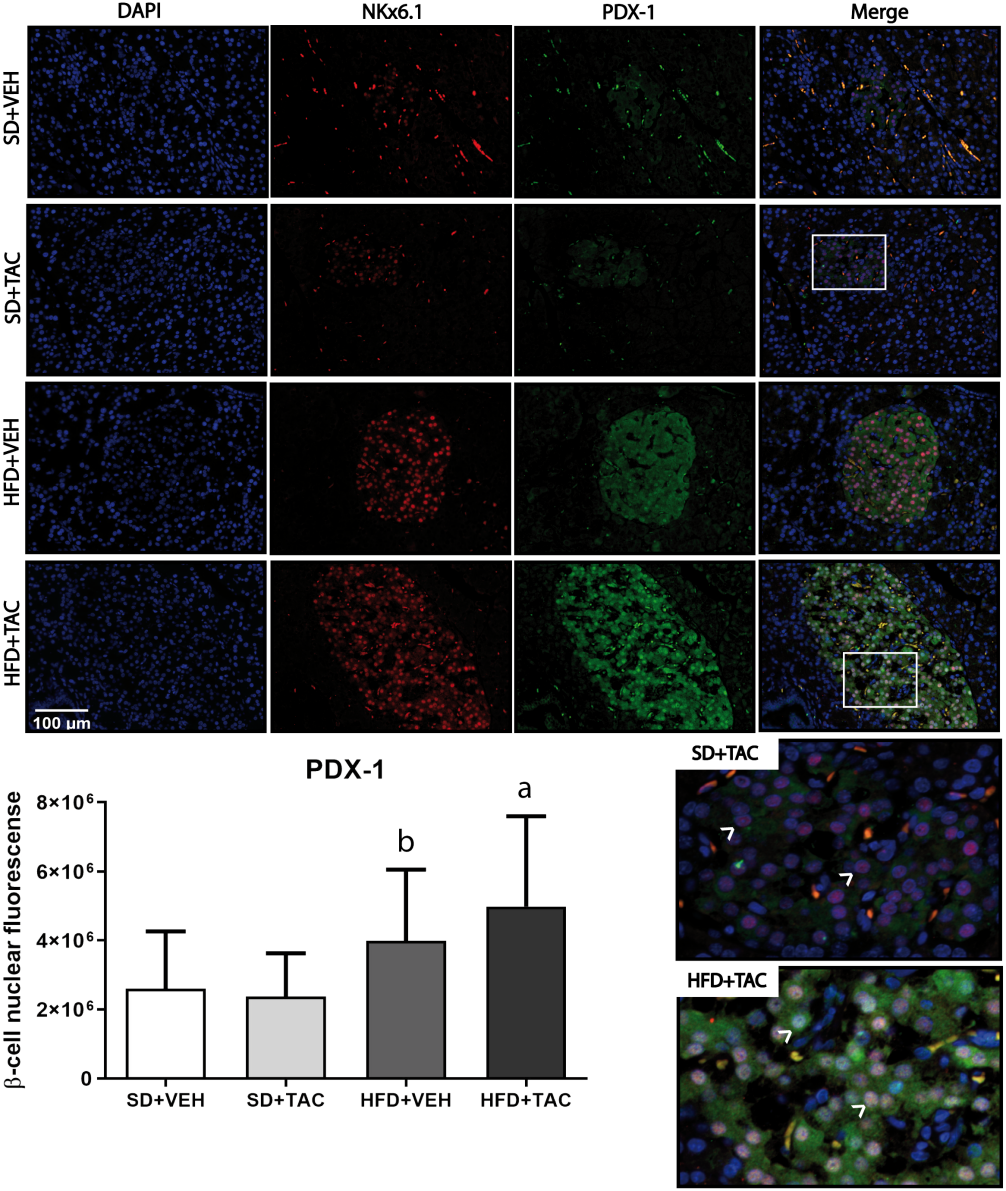
Pancreatic and duodenal homeobox (PDX-1) expression by immunofluorescence staining of Langerhans islets of pancreatic tissue and quantitative analysis. There was a marked increase in the expression of nuclear pancreatic and duodenal homeobox 1 (PDX-1) of beta cells in pancreas of both HFD+TAC and HFD+VEH group. Data are expressed as mean plus or minus standard deviation. White arrows point the lack of expression of nuclear PDX-1 in SD+TAC and the overexpression in HFD+TAC (a - HFD+TAC vs. HFD+VEH p ≤ 0.0066; HFD+TAC vs. SD+TAC p ≤ 0.0001; HFD+TAC vs. SD+VEH p ≤ 0.0001; b - HFD+VEH vs. SD+VEH p ≤ 0.0001; HFD+VEH vs. SD+TAC p ≤ 0.0001).

**Figure 5:**
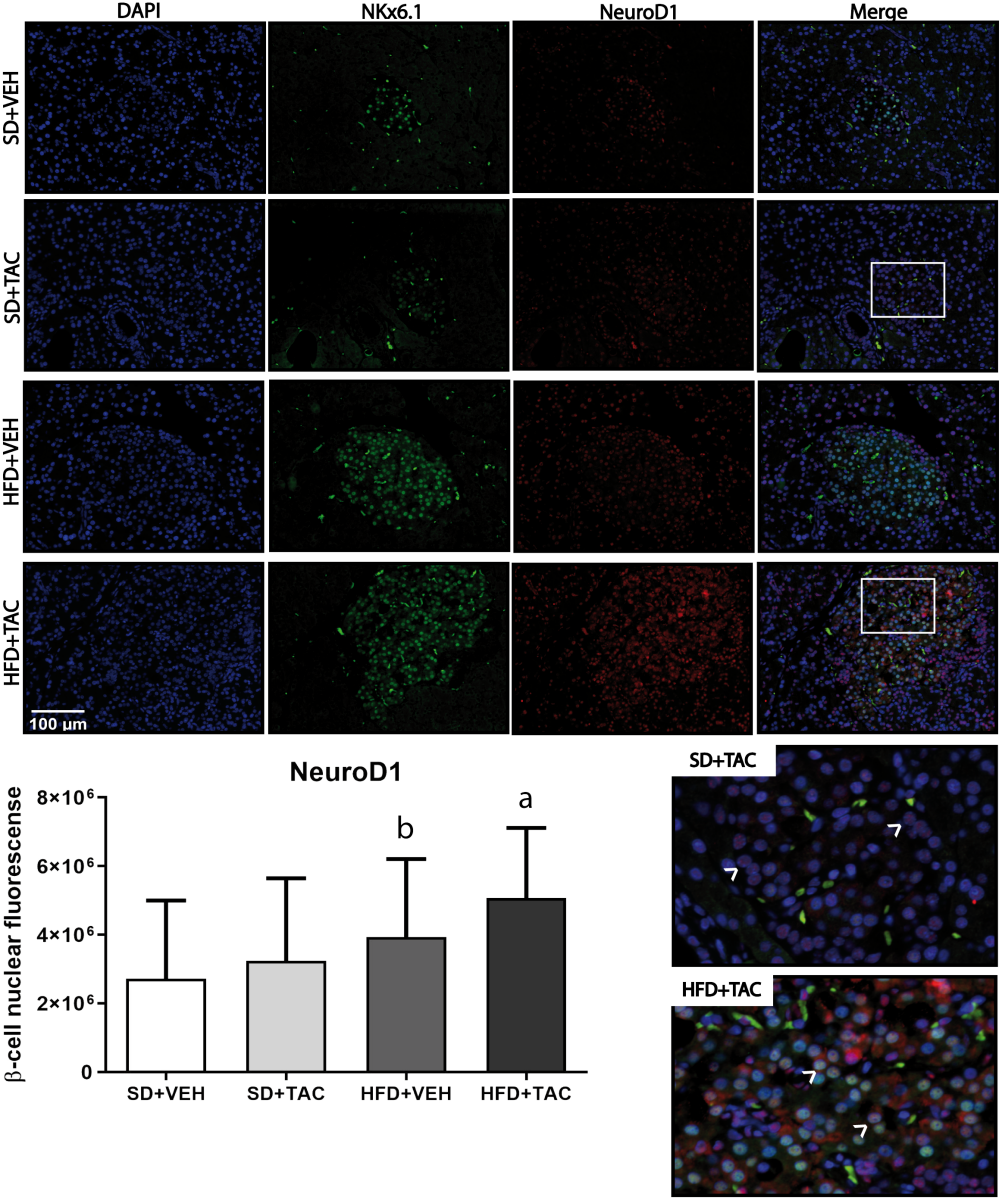
Neuronal differentiation 1 (NeuroD1) expression by immunofluorescence staining of Langerhans islets of pancreatic tissue and quantitative analysis. There was a marked increase in the expression of nuclear neuronal differentiation 1 (NeuroD1) of beta cells in pancreas of both HFD+TAC and HFD+VEH group. Data are expressed as mean plus or minus standard deviation. White arrows point the lack of expression of nuclear NeuroD1 in SD+TAC and the overexpression in HFD+TAC (a - HFD+TAC vs. HFD+VEH p ≤ 0.01; HFD+TAC vs. SD+TAC p ≤ 0.0001; HFD+TAC vs. SD+VEH p ≤ 0.0001; b - HFD+VEH vs. SD+VEH p ≤ 0.0042).

**Figure 6:**
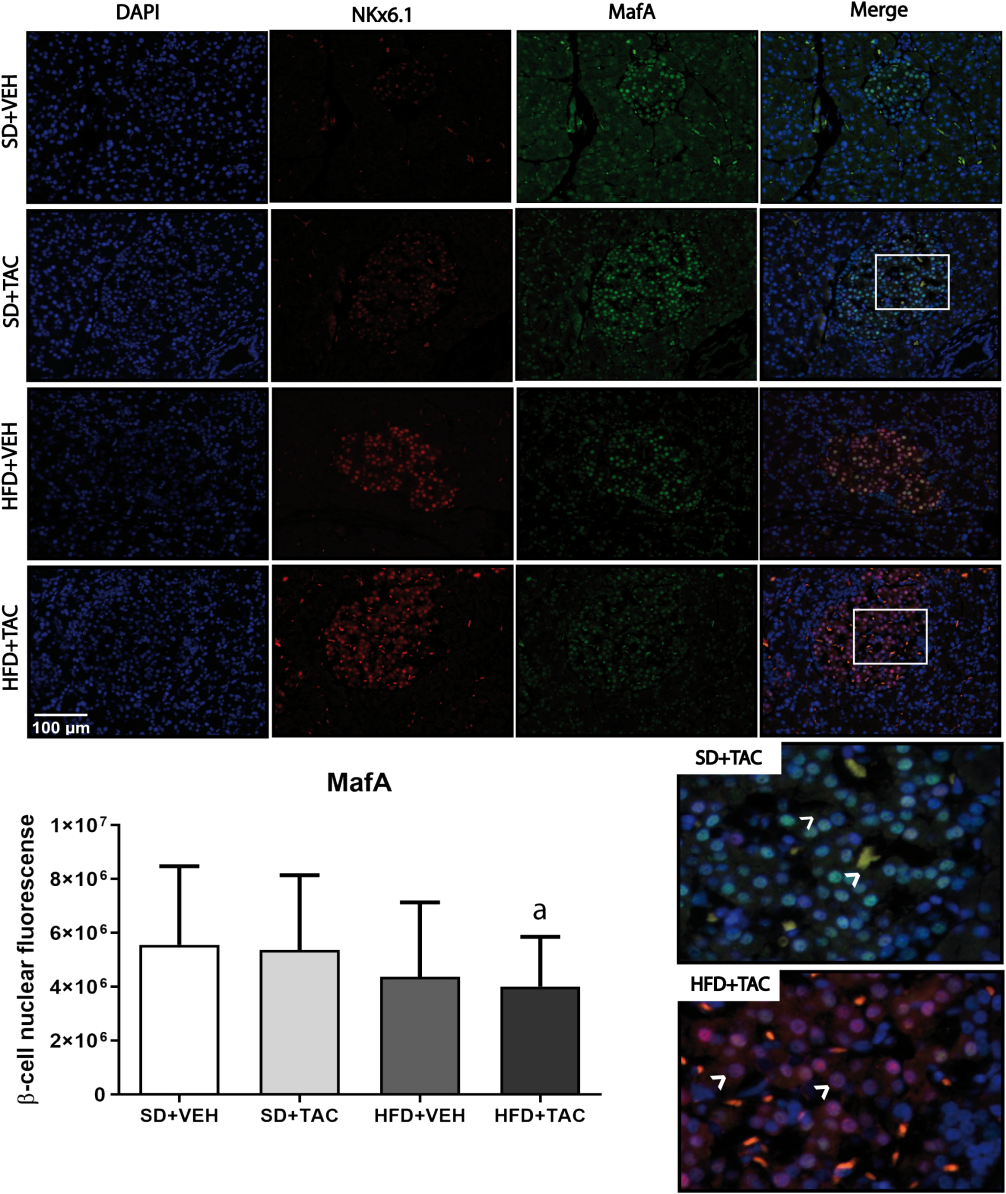
V-maf musculoaponeurotic fibrosarcoma oncogene homolog A (MafA) nuclear expression by immunofluorescence staining of Langerhans islets of pancreatic tissue and quantitative analysis. There was a clear decrease in nuclear v-maf musculoaponeurotic fibrosarcoma oncogene homolog A (MafA) of beta cells in pancreas of HFD+TAC group. Data are expressed as mean plus or minus standard deviation (a – HFD+TAC vs. SD+TAC p ≤ 0.04; HFD+TAC vs. SD+VEH p ≤ 0.02).

Animals on HFD+TAC presented an increase in nuclear FoxO1A (p ≤ 0.0001), PDX-1 (p ≤ 0.0006) and NeuroD1 (p ≤ 0.01) compared to animals on HFD+VEH (**Figures 3**–**5**). Also, animals on HFD+TAC presented lower nuclear MafA (p ≤ 0.04) compared to SD+TAC (**Figure 6**).

#### 3.4.3 Insulin and glucagon expression

**Figure 7:**
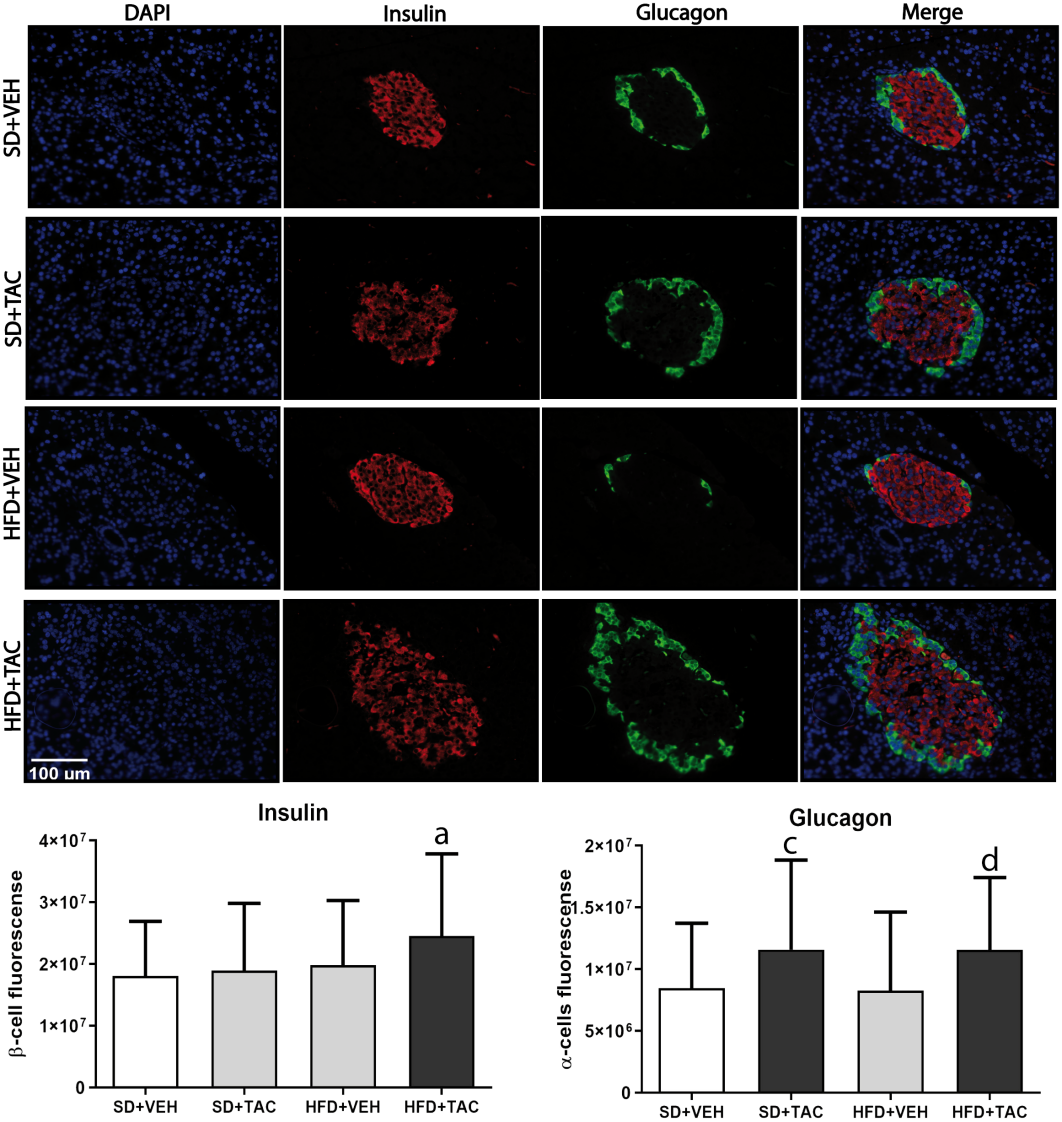
Immunofluorescence staining of insulin and glucagon in Langerhans islets of Sprague Dawley rat pancreatic tissue and quantitative analysis. There was an increase in insulin staining in HFD+TAC group. There was also an increase in glucagon staining related to an augment in alpha cells in the HFD+TAC and SD+TAC group. Data are expressed as mean plus or minus standard deviation (a – HFD+TAC vs. HFD+VEH p ≤ 0.0032; HFD+TAC vs. SD+TAC p ≤ 0.01; HFD+TAC vs. SD+VEH p ≤ 0.012; c – SD+TAC vs. SD+VEH p ≤ 0.04; SD+TAC vs. HFD+VEH p ≤ 0.0053; d – HFD+TAC vs. HFD+VEH p ≤ 0.0038; HFD+TAC vs. SD+VEH p ≤ 0.035).

Animals on HFD+TAC had higher expression of insulin compared to every other group (p ≤ 0.03) (**Figure 7**). Also, the expression of glucagon increased in HFD+TAC (p ≤ 0.0038) and in SD+TAC (p ≤ 0.0035) compared to HFD+VEH and SD+VEH respectively. The proportion of beta cells in HFD+TAC was lower than in HFD+VEH (78.6 ± 7.12% vs. 87.93 ± 8.60% p ≤ 0.0001); and in SD+TAC compared to SD+VEH (74.58 ± 11.15% vs. 85.23 ± 8.12% p ≤ 0.0001). Additionally, animals on TAC, either on HFD (21.64 ± 7.12%) or SD (25.37 ± 11.15%) had higher levels of alpha cells compared to HFD+VEH (12.07 ± 8.12%) and SD+VEH (14.77 ± 8.60%) (p ≤ 0.0001) (**Supplementary Figure 4B**).

#### 3.4.4 Proliferation and apoptosis

**Figure 8:**
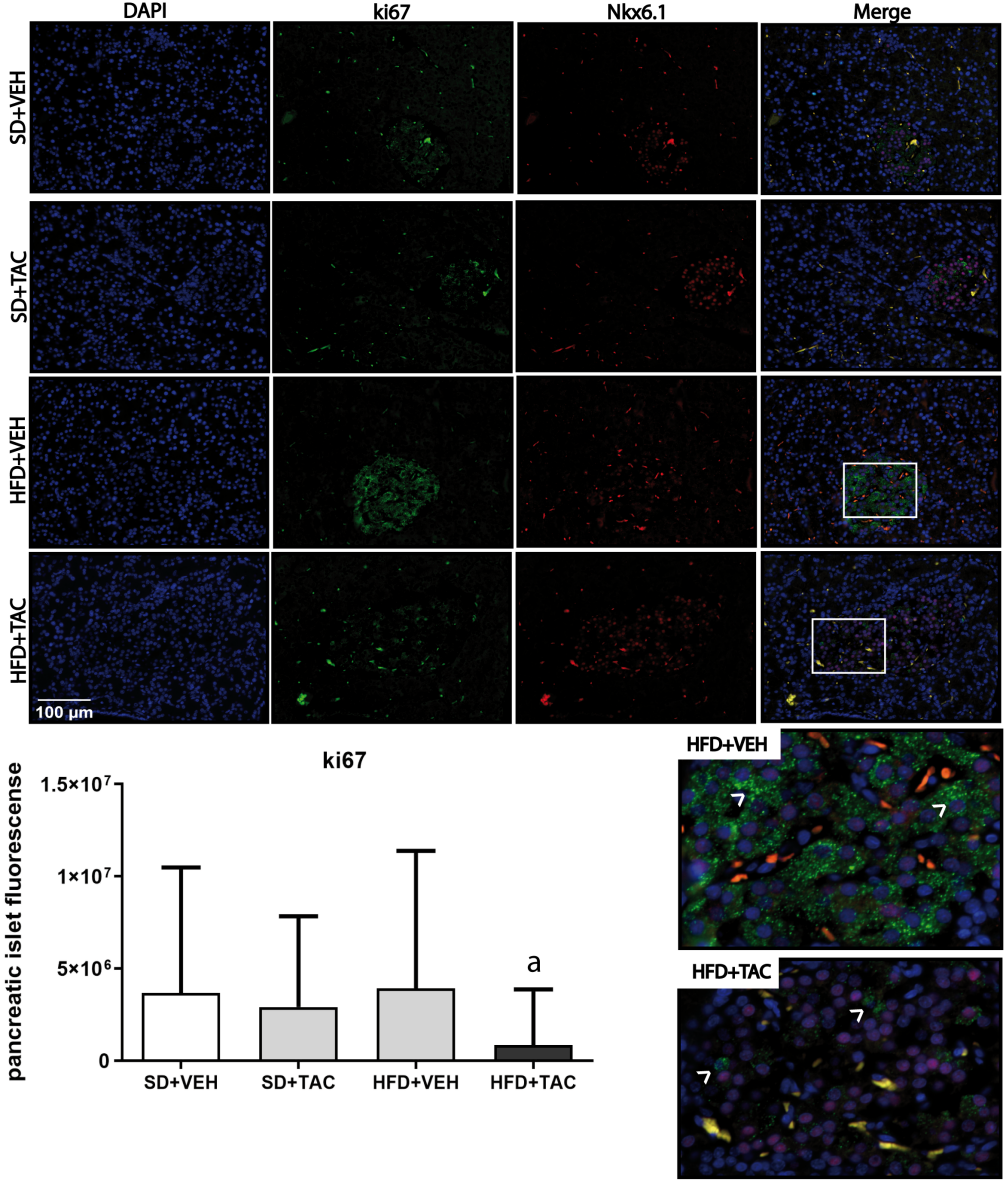
Ki67 expression by immunofluorescence staining of Langerhans islets of pancreatic tissue and quantitative analysis. There was a marked increase in the expression of ki67 in the HFD+VEH animals and a decrease in proliferation in islet of animals in HFD+TAC group. Data are expressed as mean plus or minus standard deviation. White arrows point the overexpression of Ki67 in HFD+VEH and the decreased expression in HFD+TAC (a – HFD+TAC vs. HFD+VEH p ≤ 0.0001; HFD+TAC vs. SD+TAC p ≤ 0.0072; HFD+TAC vs. SD+VEH p ≤ 0.0005).

There was a decrease in islet proliferation, determined by Ki67, in animals on HFD+TAC (p ≤ 0.0001) compared with HFD+VEH and SD+TAC animals (p ≤ 0.0072) (**Figure 8**). Regarding apoptosis, there was no signal registered of caspase 3 in Langerhans’ islets of every group (data not shown).

## 4 Discussion

In this study we developed a non-genotypic rat model of diabetes induced by Tacrolimus in the context of metabolic syndrome. Specifically, the administration of 1 mg/kg of Tacrolimus during 15 days in rats with established obesity and metabolic syndrome, induced hyperglycaemia and changes in specific beta cell nuclear factors like MafA, FoxO1A, PDX-1, NeuroD1, insulin and glucagon and reduced beta cell proliferation with no signs of apoptosis. No major changes were observed in lean animals treated with Tacrolimus.

To develop this model, we have followed previous studies of our group showing that Tacrolimus induced diabetes only in Obese Zucker rats – a genotype model of obesity and metabolic syndrome (1). In the present study, we used a different model where obesity and metabolic syndrome are induced by a fat-enriched diet. Overall, this model is more comprehensive of the pathogenesis of metabolic syndrome, a precondition of TAC-induced diabetes. Animals were fed high fat-enriched diet (HFD) during 45 days (16). On top of that, 1 mg/kg of Tacrolimus was administered during 15 days to induce diabetes. In order to confirm the development of diabetes we used gold standard methods such as intraperitoneal glucose tolerance test and insulin tolerance test.

Our main finding was that Tacrolimus promoted diabetes only in rats with obesity and metabolic syndrome, both conditions achieved by feeding the animals with an obesogenic diet. The pathogenic background of these changes could be diverse alterations in nuclear factors like MafA, FoxO1A, PDX-1 and NeuroD1 crucial for beta-cell maintenance. During states of metabolic stress (in our case the high fat diet), beta cells present active regulation of proliferation, cell growth, insulin synthesis, and secretion. These functions are controlled by transcriptional factors essential for beta cells such as MafA, FoxO1, PDX-1 and NeuroD1 (7). MafA, PDX-1 and NeuroD1 bind directly to the insulin gene promoter to induce insulin synthesis under hyperglycaemic conditions (7, 20). However, if the hyperglycaemia and insulin resistance extend over time, beta cells are unable to compensate this state and these transcriptional factors are eventually affected (7). It has been shown that the loss of MafA is an early indicator of β-cell inactivity due to hyperglycaemia, which precedes the reduction in PDX-1 and NeuroD1 for the development of diabetes (21). Tacrolimus is a potent immunosuppressive agent which exerts its action by biding to its cytoplasmic receptor: FKBP12. FKBP12 is a ubiquitous protein that may affect several intracellular pathways like transcriptional factor changes related to insulin synthesis and Ca2+ ryanodine receptors channel (RyRs). In this context, we have hypothesized that beta cells are in a compensatory state due to hyperglycaemia in which TAC accelerates the dysregulation of transcriptional factors like MafA. Besides, TAC could also compete with RyR for FKBP12 and deregulates the intracellular calcium mobilization, impairing the secretion of insulin vesicles (22) (**Supplementary Figure 5**).

Specifically, in this model we have found a decrease in nuclear MafA and an increase in nuclear FoxO1A, PDX-1 and NeuroD1 in diabetic animals. Part of these results are in line with our previous studies in Obese Zucker rats (1). In both genotypic (7) and phenotypic models of obesity, Tacrolimus induced a decrease in nuclear MafA and an increase in nuclear FoxO1A. The latter is a key transcriptional factor in the regulation of insulin and glucose homeostasis in response to stress like hyperglycaemia, glucolipotoxicity, oxidative stress or insulin resistance (7, 23, 24). FoxO1A plays an early role in beta-cell dysfunction and in the regulation of main transcription factors like MafA, PDX1 and NeuroD1 (25). In hyperglycaemia, FoxO1A could be acetylated resulting in its nuclear localization. This effect contributes to the induction of NeuroD1 and MafA augmenting insulin synthesis/secretion as a mechanism of compensation (21). Nevertheless, under metabolically stressful conditions like hyperglycaemia or lipotoxicity, it has been shown that MafA expression paradoxically decreases (7). MafA is one of the transcriptional factors essential for beta cell maturation and glucose responsiveness of adult beta cells (26). Thus, a decreased nuclear expression of MafA could lead to a beta cell dysfunction and, eventually, a progression towards diabetes (7). In our model, the gradual loss of MafA in HFD+TAC animals could be an early indicator of beta cells damage. Nevertheless, and contrary to our previous findings in Obese Zucker rats (7), animals on HFD+TAC also showed an increase in nuclear NeuroD1, nuclear PDX-1 and an increase in the relative area of the islets of Langerhans. In particular, PDX-1 has been associated to the regulation of beta cell size as a compensatory response to a stress such as a high fat enriched diet (27, 28). During beta-cell compensation, beta cell hypertrophy and hyperplasia occur to increase beta-cell mass in response to hyperglycaemia in diabetogenic states. Thus, the increase in PDX-1 in obese animals with TAC may be related with the increase in islet size considered as a compensatory response to restore the cell physiology (28). NeuroD1 is a transcription factor crucial for pancreatic development, beta cell maturation and the expansion of the pancreatic islet cell mass (29). NeuroD1 is also related to insulin signalling regulation (29) and was overexpressed in animals on HFD+TAC. This finding may be associated with the overproduction of non-secreted insulin observed in the pancreatic beta cells and the increase in islet area, possibly as a compensatory mechanism to overt hyperglycaemia and IR (30). However, it seems that this overproduction of insulin in the beta cells has not been secreted. Animals in HFD+VEH showed a marked serum hyperinsulinemia, due to obesity and high enriched fat diet (31). Nevertheless, these higher levels of serum insulin are decreased when we administered TAC on top of obesity. This could be related to some evidence which suggests that TAC could affect insulin granule of exocytosis by the impairment in Ca^2+^ channels (32), but this theory needs further investigation. Moreover, animals on HFD+TAC also had higher levels of glucagon and an increase in the proportion of alpha cells. This result is in line with the increase in the proportion of alpha cells observed in diabetic patients (33, 34). Altogether, these findings could indicate that beta cells of HFD+TAC animals are in a compensatory stage with a reduced proliferative capacity in which transcriptional factors related to insulin production and beta cells hypertrophy are overexpressed to cope with the status of hyperglycaemia and IR. Clearly, this is a short model and these changes may indicate early adaptive defensive mechanisms to glucolipotoxicity. Long-term models to evaluate chronic changes are needed to test the effect of these changes on follow-up.

Altogether, these results are complementary to our previous studies in Zucker rats and could be described as an “enriched” model which reflects more accurately the pathophysiology of diabetes. This model is adding new pathogenic changes like PDX-1, NeuroD1 and the increment of alpha cells, to those already known in transcriptional factors (MafA and FoxO1A). Previous studies have shown that PDX-1 and NeuroD1 are affected in genotypic animal models of diabetes (29, 35–37), and there is also an increase in glucagon and alpha cells in pancreatic islets (38). To our knowledge, this is the first study that shows modification of these characteristics by the interaction of obesity, metabolic syndrome and Tacrolimus. These findings are in line with studies evaluating the pathogenesis of type-2 diabetes mellitus in the general population (26, 39–43). This may be considered as evidence linking PTDM and type 2 diabetes mellitus, and deserves more attention in future studies.

This study presents some limitations. It is a short-time rodent model in which it is necessary to perform a long-term administration of Tacrolimus to evaluate potential changes in transcriptional factors and islet morphometry. In addition, it is only performed in male animals. Although there may not be predicted sex-differences in beta-cell signalling and response, they should be considered in future experimental design. Another possible limitation is the use of non-matching diet regimens. Some studies suggest the use of a Low-Fat Diet (LFD) as a more appropriate control for HFD (44, 45). Nevertheless, it has been showed that, in terms of impaired glucose tolerance, both control groups (SD and LFD) can be considered comparable and reliable (45–47). However, this could be a more realistic non-genotypic model of diabetes which could be used long term with dose regulation as a phenotypical model of chronic type 2 diabetes mellitus.

In conclusion, in our study we have induced diabetes in a phenotypic model of established obesity and metabolic syndrome using Tacrolimus as a catalyser. In these animals we obtained characteristics that resemble the physiopathology of diabetes like severe hyperglycaemia, increased size of islets of Langerhans and reduced beta-cell proliferation without changes in apoptosis. We also found changes in beta-cell nuclear factors like MafA, FoxO1A, PDX-1 and NeuroD1 and insulin and glucagon levels. Altogether, this may be instrumental to study the pathogenesis of type 2 diabetes mellitus.

## Data availability statement

The raw data supporting the conclusions of this article will be made available by the authors, without undue reservation.

## Ethics statement

The animal study was approved by Animal Care and Use Committee (Comité de Ética de la Investigación y de Bienestar Animal (CEIBA) of University of La Laguna, Spain). The study was conducted in accordance with the local legislation and institutional requirements.

## Author contributions

ST-T: Writing – review & editing, Writing – original draft, Visualization, Validation, Supervision, Software, Resources, Project administration, Methodology, Investigation, Funding acquisition, Formal analysis, Data curation, Conceptualization. EP: Writing – review & editing, Writing – original draft, Visualization, Validation, Supervision, Software, Resources, Project administration, Methodology, Investigation, Funding acquisition, Formal analysis, Data curation, Conceptualization. LM-Q: Writing – review & editing, Writing – original draft, Validation, Methodology, Formal analysis, Data curation. AT-R: Conceptualization, Writing – review & editing, Writing – original draft, Supervision, Methodology. CF: Writing – review & editing, Writing – original draft, Resources, Methodology. AR-R: Writing – review & editing, Writing – original draft, Visualization, Validation, Supervision, Software, Resources, Project administration, Methodology, Investigation, Funding acquisition, Formal analysis, Data curation, Conceptualization.
