# Supplementary material for: Induction of diabetes by Tacrolimus in a phenotypic model of obesity and metabolic syndrome
Source: Front Endocrinol (Lausanne). 2024 Apr 29;15:1388361. doi: 10.3389/fendo.2024.1388361 (PMC11092379; doi:10.3389/fendo.2024.1388361)
Supplement: Supplementary file 1 [file DataSheet_1.docx]

Supplementary Material

# Supplementary Figures and Tables

## Supplementary Figures


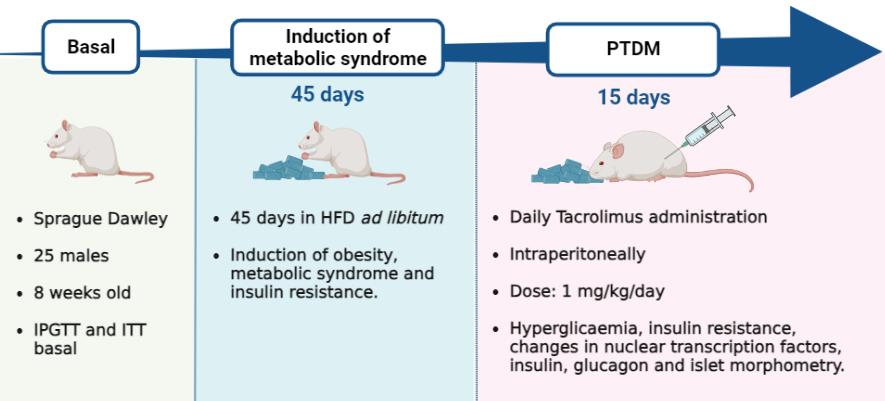


**Supplementary figure 1. Protocol for the induction of diabetes with Tacrolimus in a phenotypic model of obesity and metabolic syndrome.** 25 Sprague Dawley male rats of approximately 8 weeks old were included in the experimental design. 14 rats were fed by 45 days with a high fat diet ad libitum to induce obesity and metabolic syndrome. After this period, 7 rats were administered daily with 1 mg/kg of Tacrolimus to accelerate the development of diabetes. At the end of the treatments, animals presented diabetes-like characteristic as hyperglycaemia, insulin resistance and changes in nuclear factors, insulin, glucagon and islet morphometry**.**


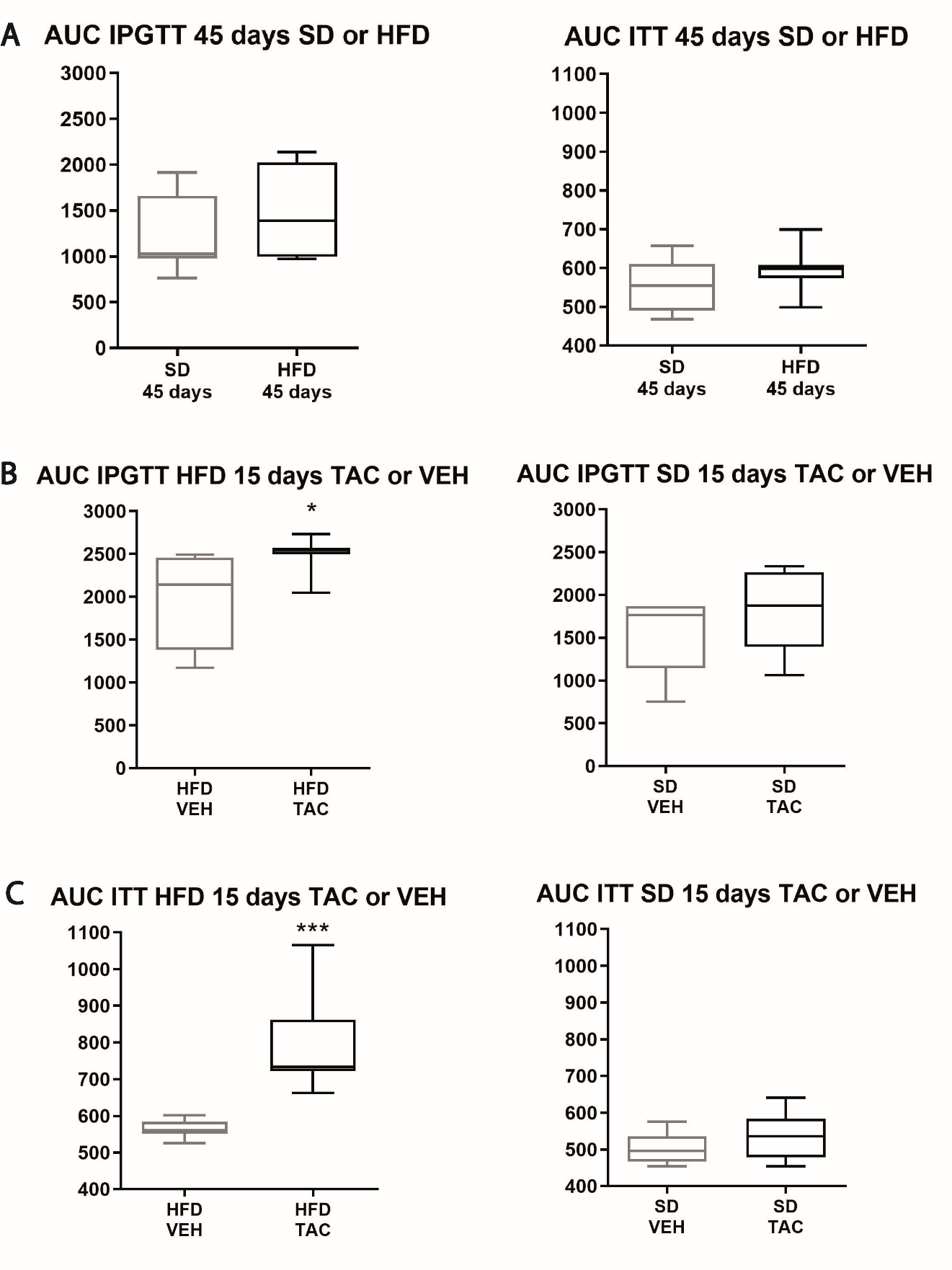


**Supplementary figure 2. Area under the curve of intra-peritoneal tolerance test and insulin tolerance tests. A.** AUC of glycaemias in IPGTT and ITT at 45 days of HFD or SD. **B.** AUC of glycaemias in IPGTT after 15 days of TAC or VEH administration in HFD and SD (p≤0.0011). **C.** AUC of glycaemias in ITT after 15 days of TAC or VEH administration in HFD and SD (p≤0.0006). Data are expressed as mean plus or minus standard deviation.


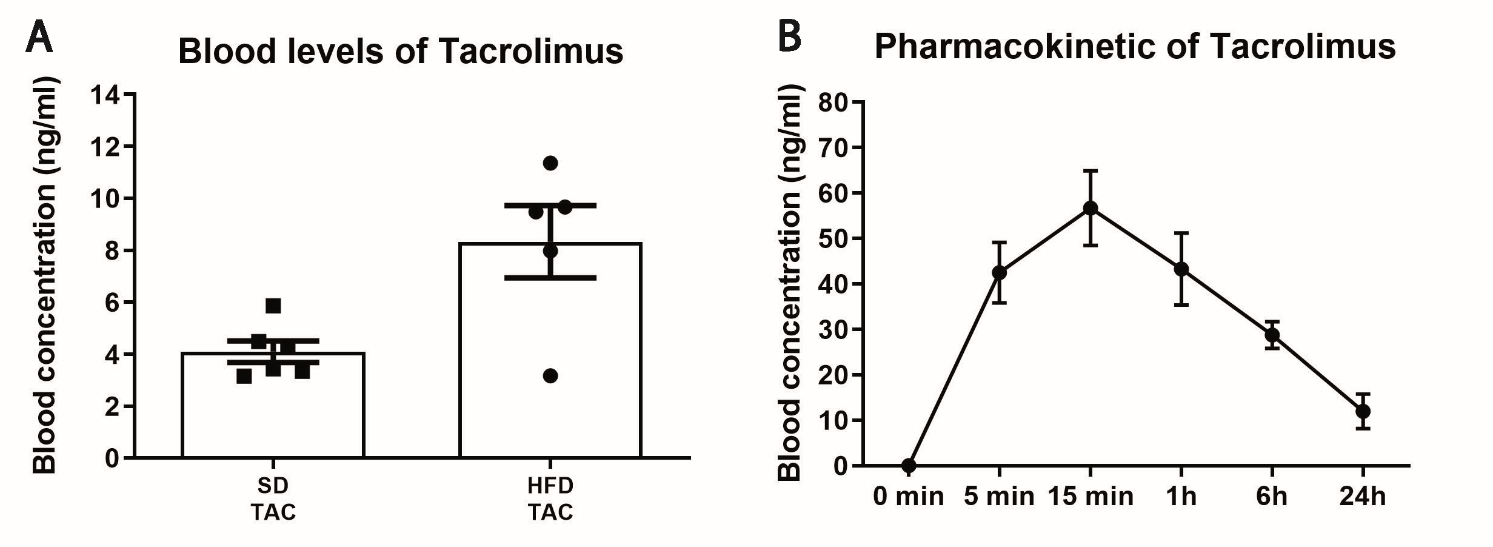


**Supplementary figure 3. Blood levels of Tacrolimus at endpoint and Tacrolimus pharmacokinetic. A.** Blood levels of Tacrolimus at endpoint in HFD (8.33 ± 3.1 ng/mL) and SD group (4.16 ± 1.1 ng/mL). **B.** Pharmacokinetic of 1 mg/kg of Tacrolimus. Data are expressed as mean plus or minus standard deviation.


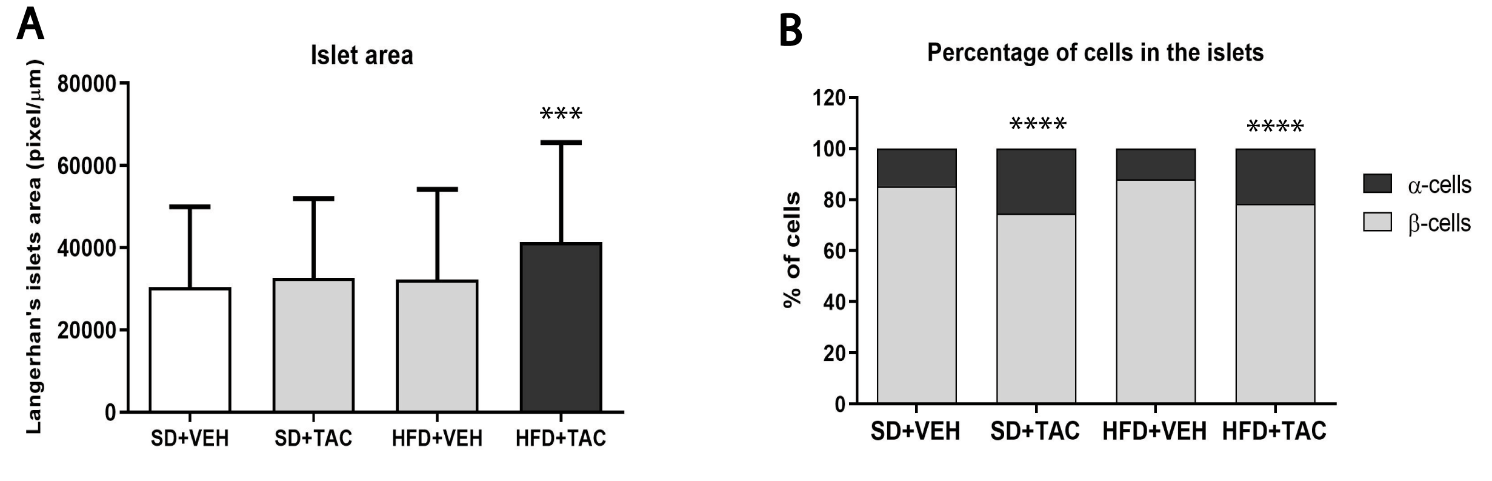


**Supplementary figure 4. Morphometric analysis of Langerhans islets. A.** Langerhans islet area of each group (HFD+TAC p≤0.0005 with the other groups). **B.** Percentage of alpha and beta cells in the islets of each group. Increase in the proportion of alpha cells and decrease in the proportion of beta cells in HFD+TAC and SD+TAC animals. (HFD+TAC vs. HFD+VEH p≤0.0001 and SD+TAC vs. SD+VEH p≤0.0001). Data are expressed as mean plus or minus standard deviation.

**
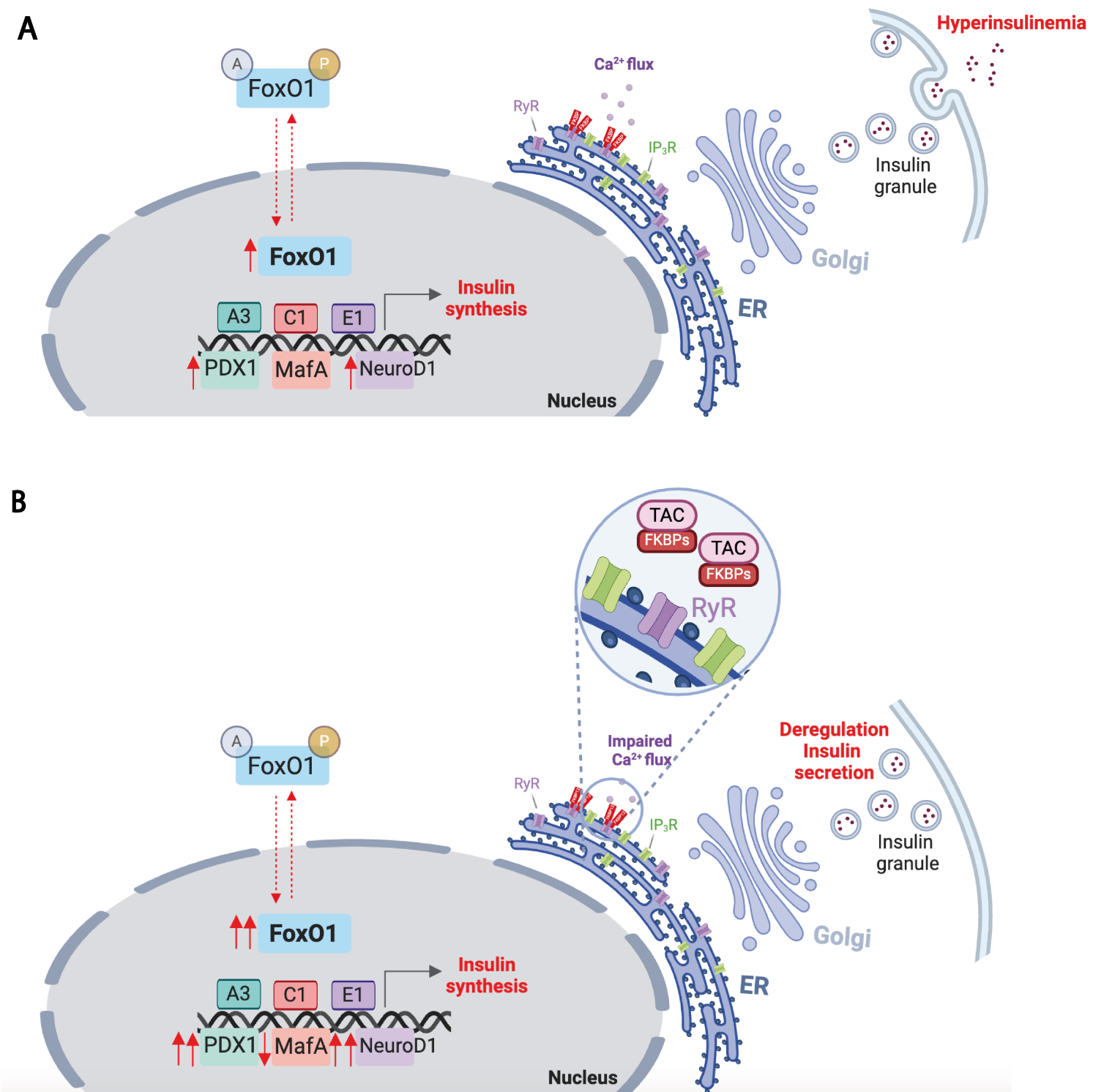
**

**Supplementary figure 5. Schematic illustration of the action of Tacrolimus in the context of glucolipotoxicity on pancreatic β-cell. A.** During glucolipotoxicity, β-cells are able to compensate by activating proliferative and cell growth pathways and enhancing insulin synthesis and secretion. This compensation state can be controlled in part by the upregulation of essential transcriptional factors like FoxO1, MafA, PDX-1 and NeuroD1. Moreover, the insulin vesicles are actively secreting which leads to a marked hyperinsulinemia. **B.** Tacrolimus (TAC) binds to their cytoplasmic receptor FKBP12 to exert its action. TAC, on top of glucolipotixicity, is able to induce the overexpression of PDX-1 and NeuroD1 and accelerates the dysregulation of transcriptional factor MafA. The loss of MafA is an early indicator of β-cell inactivity due to hyperglycaemia, which precedes the reduction in PDX-1 and NeuroD1 for the development of diabetes. TAC also competes with Rianodine Receptor (RyR) for FKBP12 and is able to deregulates intracellular calcium mobilization, impairing the secretion of insulin vesicles.
